# Construction and validation of a predictive model for new-onset atrial fibrillation in patients with acute myocardial infarction following emergency percutaneous coronary intervention based on novel inflammatory markers

**DOI:** 10.3389/fcvm.2025.1718098

**Published:** 2026-01-12

**Authors:** Yang Yang, Hai Xu, Xiuyu Liang, Zheng Gong, Leilei Liu, Jingjing Yuan, Yongsheng Wang, Xiaohong Zhang

**Affiliations:** Department of Cardiology, The First People’s Hospital of Hefei, The Third Affiliated Hospital of Anhui Medical University, Hefei, Anhui, China

**Keywords:** AMI, new-onset AF, novel inflammatory markers, predictive model, systemic inflammatory response index

## Abstract

**Objective:**

Inflammatory biomarkers are established predictors of outcomes in cardiovascular diseases, yet their accuracy in predicting new-onset atrial fibrillation (NOAF) after emergency percutaneous coronary intervention (PCI) in patients with acute myocardial infarction (AMI) remains uncertain. This study aimed to evaluate the predictive value of emerging inflammatory indices for NOAF following PCI in AMI patients and to develop a clinically applicable nomogram.

**Methods:**

A retrospective analysis was performed on 509 AMI patients who had no prior history of atrial fibrillation. Univariate and multivariate logistic regression analyses were utilized to determine significant preoperative inflammatory biomarkers and other clinical risk factors associated with NOAF. A predictive nomogram was subsequently created based on these identified factors. The discriminative ability, accuracy of calibration, and clinical utility of the nomogram were evaluated through receiver operating characteristic (ROC) curves, calibration plots, and decision curve analysis (DCA).

**Results:**

Among the 509 patients studied, 94 (18.5%) experienced NOAF during hospitalization. Multivariate logistic regression analyses revealed that advanced age, increased left atrial diameter (LAD), higher Killip class, log-transformed NT-proBNP and increased systemic inflammatory response index (SIRI) independently predicted NOAF risk among AMI patients after PCI. ROC analyses comparing several novel inflammatory indicators, neutrophil-to-lymphocyte ratio (NLR), lymphocyte-to-monocyte ratio (LMR), platelet-to-lymphocyte ratio (PLR), and SIRI—demonstrated that SIRI exhibited the highest predictive accuracy for NOAF occurrence (AUC: 0.813, 95% CI: 0.739–0.887). Additionally, a nomogram including SIRI was constructed to predict the risk of in-hospital NOAF in AMI patients. The C-index, equivalent to the area under the ROC curve (AUC), was 0.868 (95% CI: 0.821–0.916) in the training cohort and 0.859 (95% CI: 0.793–0.925) in the validation cohort, indicating good discrimination. Calibration plots confirmed good agreement between predicted probabilities and observed outcomes, and decision curve analysis verified substantial clinical benefit.

**Conclusion:**

SIRI was identified as the most effective inflammatory biomarker for predicting NOAF in AMI patients following PCI. The constructed nomogram, which incorporates inflammatory indicators, allows rapid and personalized assessment, enabling clinicians to better identify and manage AMI patients at increased risk for NOAF.

## Introduction

AMI remains a significant public health challenge globally, linked to elevated mortality rates worldwide (1). Although PCI effectively restores coronary perfusion, reperfusion injury can aggravate myocardial damage, increasing susceptibility to cardiac arrhythmias. Atrial fibrillation (AF) frequently develops in AMI patients, with reported prevalence ranging between 6% and 21% (2). In STEMI patients specifically, the incidence of postoperative NOAF following PCI ranges from 3.0% to 13.7% (3, 4). The development of NOAF during hospitalization in AMI patients is linked with extended hospitalization durations, elevated morbidity, and increased mortality rates, ultimately resulting in adverse clinical outcomes (5, 6). Despite various factors being associated with NOAF initiation and progression, the exact mechanisms have yet to be fully clarified. Thus, prompt identification and intervention for AMI patients at heightened NOAF risk are crucial to enhancing outcomes and survival rates. Creating reliable and effective predictive tools can significantly assist clinicians in timely risk assessment, early detection, and targeted intervention in AMI patients susceptible to NOAF after emergency PCI.

Numerous studies have demonstrated a close relationship between inflammatory response and AF. The occurrence of AF results from necrosis and fibrosis induced by inflammatory processes, and atrial remodeling during inflammation provides a foundation for the initiation and persistence of AF (7). Various structural changes in the atria, including atrial dilation, hypertrophy of atrial cardiomyocytes, and fibrosis, may lead to shortened action potentials, reduced electrical coupling between cells, and abnormal calcium handling. These changes directly trigger atrial arrhythmias through membrane potential fluctuations (8). MI can induce atrial inflammation and elevate the risk of developing AF (9, 10). Therefore, assessing inflammatory status may be valuable in predicting AF occurrence in AMI patients. Recently, parameters derived from complete blood count (CBC), including NLR, LMR, PLR, and SIRI, have been used as markers to evaluate systemic inflammatory status (11–14). These parameters have demonstrated value as inflammatory biomarkers for AF and can be readily obtained from blood samples through routine laboratory testing, with the advantages of low cost and high reproducibility. However, the predictive value of these four novel inflammatory markers for NOAF in AMI patients after PCI has not yet been fully investigated.

Therefore, this study aims to evaluate the predictive value of preoperative NLR, LMR, PLR, and SIRI for post-PCI NOAF in AMI patients. In addition, a simplified nomogram incorporating inflammatory markers was developed to assist clinicians in predicting NOAF occurrence among AMI patients, thereby optimizing clinical management.

## Methods

### Study population

**Figure 1:**
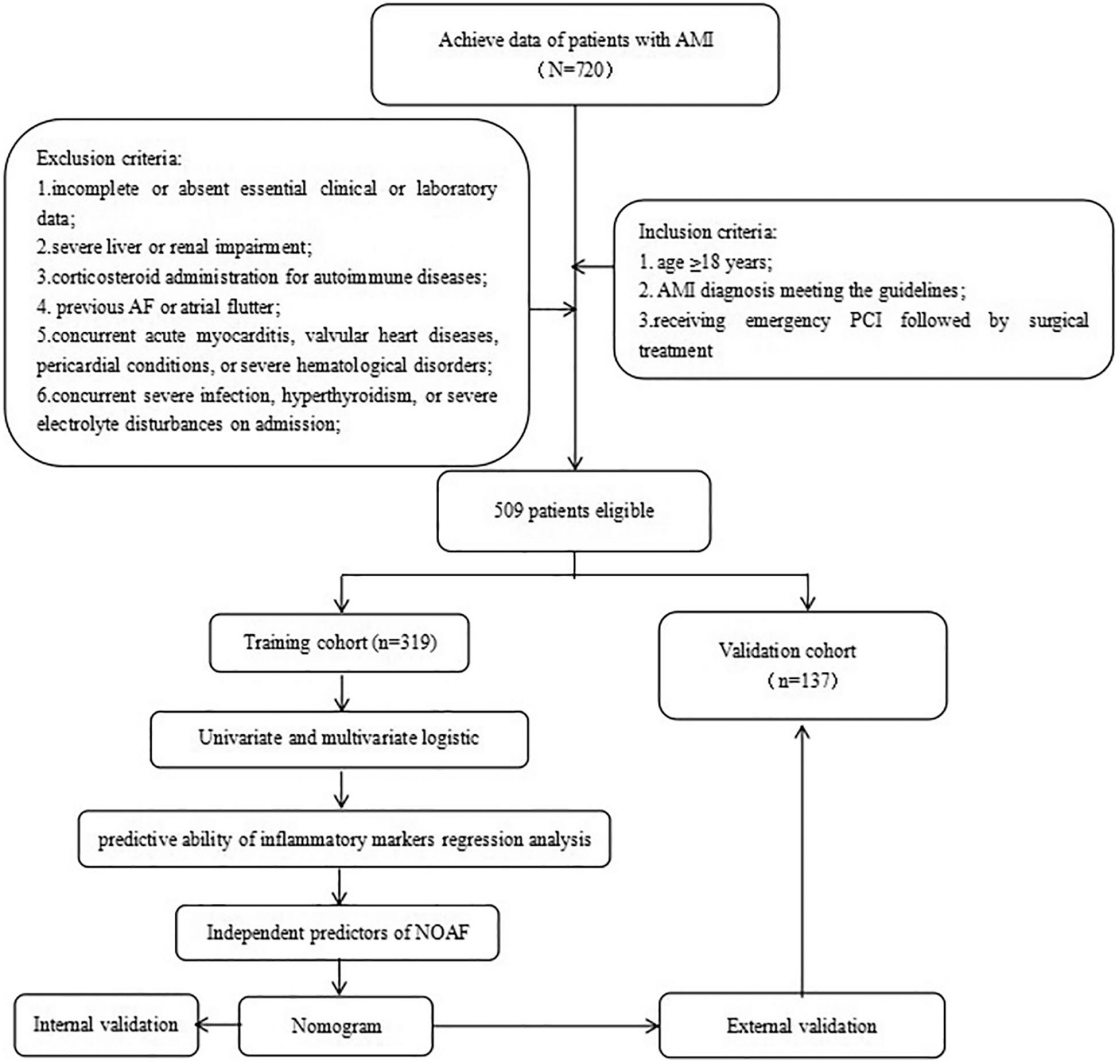
Summary of patient flow diagram. AMI, acute myocardial infarction; PCI, percutaneous coronary intervention; NOAF, new-onset atrial fibrillation.

This study retrospectively recruited 720 AMI patients admitted to the Third Affiliated Hospital of Anhui Medical University between March 2019 and March 2024. Inclusion criteria: (1) age ≥18 years; (2) AMI diagnosis meeting the guidelines (15); (3) receiving emergency PCI followed by surgical treatment. Exclusion criteria included: (1) incomplete or absent essential clinical or laboratory data; (2) severe liver or renal impairment; (3) corticosteroid administration for autoimmune diseases; (4) previous AF or atrial flutter; (5) concurrent acute myocarditis, valvular heart diseases, pericardial conditions, or severe hematological disorders; (6) concurrent severe infection, hyperthyroidism, or severe electrolyte disturbances on admission. After applying these criteria, 509 patients qualified for further analysis (Figure 1). The eligible patients were randomly allocated into two subsets: a training cohort comprising 356 individuals and a validation cohort consisting of 153 individuals. Furthermore, participants were classified into NOAF (*n* = 94) and non-NOAF (*n* = 415) groups.

### Baseline data

Clinical data were retrospectively collected, including demographic information (age, sex, heart rate, systolic and diastolic blood pressure, smoking and alcohol consumption history, and medication use); laboratory parameters on admission day [neutrophil, monocyte, lymphocyte, platelet, erythrocyte counts, total cholesterol, triglycerides, very low-density lipoprotein cholesterol (VLDL-C), creatinine, uric acid, estimated glomerular filtration rate (eGFR), NT-proBNP, CK-MB, and cTnI levels]; presence of comorbidities (hypertension, diabetes mellitus, and previous history of coronary artery disease); echocardiographic parameters [left ventricular end-diastolic diameter (LVEDD), LAD, and left ventricular ejection fraction (LVEF)]; and other relevant clinical data (type of AMI, coronary artery stenosis type, Killip classification, and CHA_2_DS_2_-VASc scores). Additionally, Inflammation-derived indicators were computed with the following equations: NLR represented the ratio of neutrophil count to lymphocyte count; PLR represented the platelet count divided by lymphocyte count; LMR represented lymphocyte count divided by monocyte count; and SIRI was calculated by multiplying neutrophil and monocyte counts and then dividing the result by lymphocyte count.

### Definitions

NOAF following PCI was defined as atrial fibrillation lasting at least half a minute, detected through continuous electrocardiogram (ECG) monitoring, standard 12-lead ECG recordings, or dynamic ECG in hospitalized AMI patients with no previous history of AF (16). All patients underwent continuous ECG monitoring for at least 72 h post-PCI, supplemented by daily 12-lead ECG recordings until discharge. Significant coronary artery stenosis was identified when angiography demonstrated narrowing of 50% or greater in any coronary vessel. The Killip classification was categorized as follows: Class I represented no signs of heart failure; Class II indicated pulmonary rales in less than half the lung field; Class III indicated pulmonary rales in over half of the lung field; and Class IV denoted cardiogenic shock accompanied by diverse hemodynamic instability (17).

### Statistical analysis

Data analysis was conducted using R software (version 4.4.1). Continuous variables with normal distributions were presented as means ± standard deviations and compared between groups using independent-sample *t*-tests. Variables not conforming to normal distributions were described as medians [P25, P75]. Logarithmic transformation was applied to the non-normally distributed continuous variable NT-proBNP to normalize its skewed distribution before inclusion in the regression analysis, and comparisons between groups were conducted using the Wilcoxon rank-sum test. Categorical data were presented as frequencies (percentages), and comparisons between groups were performed using chi-square tests or Fisher's exact tests when chi-square assumptions were not met. Given the retrospective nature of the study, a formal *a priori* sample size calculation was not performed; however, *post hoc* power analysis indicated a power >0.85 for the primary predictors. Missing data accounted for <5% of variables and were handled via multiple imputation using the “missRanger” package. Sensitivity analysis comparing the original and imputed datasets showed consistent results.

Data were randomly partitioned into a training set (70%) for model development and a validation set (30%) for model assessment. Predictive model construction proceeded as follows: First, univariate logistic regression analysis was conducted to identify potential predictors (*P* < 0.05). Significant variables were subsequently entered into a multivariate logistic regression model using stepwise selection (bidirectional), and variables were assessed for multicollinearity using the variance inflation factor (VIF). After excluding variables exhibiting multicollinearity, the final predictive variables were identified to construct a nomogram. The performance of the nomogram was validated through 1,000 bootstrap resamples in both training and validation cohorts. A calibration curve was generated to evaluate model calibration, and the Hosmer–Lemeshow test was applied to assess the model's goodness-of-fit. The discriminative performance of the model was assessed using receiver operating characteristic (ROC) curve analysis, and metrics including area under the curve (AUC), sensitivity, and specificity were calculated. Additionally, decision curve analysis (DCA) was conducted to evaluate the model's clinical utility by quantifying net benefit across threshold probabilities. A two-sided *P*-value <0.05 was considered statistically significant.

## Results

### Sample characteristics

In total, 509 patients diagnosed with AMI participated in this study, and 94 patients (18.5%) experienced NOAF. Participants were randomly assigned to a training group comprising 356 individuals and a validation group consisting of 153 individuals, at a 7:3 ratio (Figure 1).

Baseline clinical and demographic characteristics for all patients (*n* = 509), the training cohort (*n* = 356), and the validation cohort (*n* = 153) are summarized in Table 1. The proportion of male patients was greater in the training group, while the incidence of Killip classification ≥II was substantially lower (*P* = 0.032). Apart from these, no significant statistical differences were observed between groups regarding age, hypertension, diabetes, prior coronary artery disease, smoking or alcohol consumption history, AMI subtypes, infarct-related artery location, echocardiographic parameters, biochemical and inflammatory markers, vital signs upon admission, CHA_2_DS_2_-VASc scores, or medication usage (all *P* > 0.05). Overall, the balance in baseline characteristics between these cohorts ensured the validity and accuracy of the subsequent analyses and model validation.

**Table 1:** Baseline characteristics of patients in the training and validation cohorts.

Variable	Overall	Training	Internal test	*P*-value
	*N* = 509	*N* = 356	*N* = 153	
Age (years)	65 (53, 75)	66 (53, 74)	63 (53, 77)	0.989
Male, *n* (%)	406 (79.76%)	294 (82.58%)	112 (73.20%)	0.016
History of hypertension, *n* (%)	274 (53.83%)	185 (51.97%)	89 (58.17%)	0.198
History of diabetes mellitus, *n* (%)	191 (37.52%)	132 (37.08%)	59 (38.56%)	0.751
History of coronary heart disease, *n* (%)	38 (7.47%)	23 (6.46%)	15 (9.80%)	0.188
Drinking, *n* (%)	88 (17.29%)	61 (17.13%)	27 (17.65%)	0.889
Smoking, *n* (%)	235 (46.17%)	165 (46.35%)	70 (45.75%)	0.901
Type of AMI, *n* (%)				0.391
NSTEMI	143 (28.09%)	104 (29.21%)	39 (25.49%)	
STEMI	366 (71.91%)	252 (70.79%)	114 (74.51%)	
Culprit vessels, *n* (%)				
LAD	263 (51.67%)	181 (50.84%)	82 (53.59%)	0.569
RCA	184 (36.15%)	128 (35.96%)	56 (36.60%)	0.889
LCX	70 (13.75%)	55 (15.45%)	15 (9.80%)	0.090
LVEDD (mm)	51.0 (48.0, 55.0)	50.0 (48.0, 54.0)	51.0 (48.0, 56.0)	0.431
LAD (mm)	38.0 (35.0, 40.0)	38.0 (35.0, 40.0)	38.0 (35.0, 40.0)	0.359
LVEF (%)	60 (53, 65)	60 (53, 65)	58 (52, 65)	0.225
cTnI (ng/mL)	17 (6, 56)	19 (6, 58)	16 (5, 55)	0.250
CK-MB (μ/L)	63 (37, 173)	64 (37, 173)	59 (37, 178)	0.719
NT-ProBNP (pg/mL)	1,042 (386, 3,450)	994.50 (417, 2,489)	993 (297, 3,434)	0.653
TG (mmol/L)	1.45 (1.02, 2.31)	1.52 (1.03, 2.31)	1.36 (0.98, 2.21)	0.313
TC (mmol/L)	4.47 (3.78, 5.26)	4.44 (3.78, 5.29)	4.55 (3.78, 5.15)	0.983
VLDL (mmol/L)	0.61 (0.42, 0.98)	0.63 (0.43, 0.97)	0.57 (0.39, 1.02)	0.584
Uric acid (μmol/L)	342 (275, 398)	342 (277, 399)	342 (274, 398)	0.583
Creatinine (μmol/L)	76 (67, 85)	76 (67, 84)	75 (62, 86)	0.417
GFR (mL/min)	91 (77, 101)	91 (78, 99)	92 (75, 104)	0.396
HGB (g/L)	134 (122, 146)	136 (123, 147)	132 (121, 143)	0.070
Neutrophil (10^9^/L)	6.99 (5.38, 9.02)	7.00 (5.35, 8.98)	6.98 (5.51, 9.11)	0.896
Lymphocyte (10^9^/L)	1.32 (0.96, 1.78)	1.34 (0.96, 1.76)	1.27 (0.96, 1.83)	0.927
Monocyte (10^9^/L)	0.53 (0.39, 0.70)	0.53 (0.39, 0.70)	0.53 (0.39, 0.70)	0.932
Platelet (10^9^/L)	200 (170, 238)	198 (170, 237)	208 (169, 246)	0.429
NLR	5.6 (3.4, 7.9)	5.6 (3.5, 8.0)	5.9 (3.4, 7.6)	0.954
PLR	154 (112, 208)	155 (112, 205)	153 (110, 220)	0.587
LMR	2.44 (1.55, 3.98)	2.41 (1.55, 3.99)	2.52 (1.53, 3.89)	0.963
SIRI	2.8 (1.6, 4.8)	2.8 (1.6, 5.0)	2.9 (1.5, 4.5)	0.837
HR (bpm)	80 (70, 90)	79 (69, 91)	80 (70, 90)	0.609
SBP (mmHg)	130 (116, 143)	130 (116, 143)	130 (117, 143)	0.844
DBP (mmHg)	78 (70, 88)	78 (70, 88)	79 (70, 88)	0.748
Killip class ≥ II, *n* (%)	165 (32.40%)	106 (29.50%)	61 (39.20%)	0.032
Medications in-hospital, *n* (%)				
ACEI/ARB	218 (42.83%)	147 (41.29%)	71 (46.41%)	0.285
Beta blocker	351 (68.96%)	248 (69.66%)	103 (67.32%)	0.600
CHA_2_DS_2_-VASc score	2.00 (1.00, 4.00)	2.00 (1.00, 4.00)	2.00 (1.00, 4.00)	0.438

NSTEMI, non-ST segment elevation myocardial infarction; STEMI, ST segment elevation myocardial infarction; LAD, left anterior descending coronary artery; LCX, left circumflex coronary artery; RCA, right coronary artery; LVEDD, left ventricular end-diastolic diameter; LAD, left atrial diameter; LVEF, left ventricular ejection fraction; CK-MB, creatine kinase-MB; cTnI, cardiac troponin I; NT-Probnp, N-terminal pro-brain natriuretic peptide; GFR, glomerular filtration rate; HGB, hemoglobin; TC, total cholesterol; TG, triglycerides; VLDL, very low-density lipoprotein; NLR, neutrophil-tolymphocyte ratio; LMR, lymphocyte-to-monocyte ratio; PLR, platelet-to-lymphocyte ratio; SIRI, systemic inflammation response index; SBP, systolic blood pressure; DBP, diastolic blood pressure.

### Risk factor analysis and selection of characteristics for NOAF after emergency PCI

Before regression analysis, the variable NT-proBNP was subjected to logarithmic transformation. Univariate logistic regression analysis identified 18 candidate variables (age, LVEDD, LAD, LVEF, log NT-proBNP, TG, VLDL, creatinine, GFR, neutrophils, monocytes, NLR, LMR, SIRI, admission heart rate, DBP, Killip classification ≥II, CHA_2_DS_2_-VASc score) significantly associated with in-hospital NOAF following PCI in patients with AMI (Table 2). Variables with *P*-values <0.05 in univariate logistic regression were subsequently incorporated into multivariate logistic regression analysis, identifying five independent predictive factors for NOAF post-PCI: age [OR = 1.014 (95% CI: 1.010–1.070), *P* = 0.006], LAD [OR = 1.100 (95% CI: 1.010–1.190), *P* = 0.020], Killip classification [OR = 2.510 (95% CI: 1.150–5.520), *P* = 0.022], log NT-proBNP [OR = 1.520 (95% CI: 1.130–2.030), *P* = 0.005], and SIRI [OR = 1.310 (95% CI: 1.180–1.460), *P* < 0.001] (Table 2). The variance inflation factors (VIF) for all variables were below 1.7, indicating no multicollinearity among the predictors.

**Table 2:** Univariate and multivariate logistic analyses to determine the independent predictors associated with NOAF in the training cohort.

Variable	Univariate analysis			Multivariate analysis		
	OR	95% CI	*P*-value	OR	95% CI	*P*-value
Age (years)	1.061	1.036, 1.087	<0.001	1.014	1.010, 1.070	0.006
Male, *n* (%)	0.754	0.380, 1.494	0.418			
History of hypertension, *n* (%)	0.983	0.568, 1.701	0.951			
History of diabetes mellitus, *n* (%)	1.505	0.864, 2.619	0.149			
History of coronary heart disease, *n* (%)	1.345	0.480, 3.771	0.573			
Drinking, *n* (%)	0.790	0.367, 1.703	0.548			
Smoking, *n* (%)	0.944	0.544, 1.636	0.837			
Type of AMI, *n* (%)						
NSTEMI			Reference			
STEMI	1.113	0.604, 2.051	0.733			
Culprit vessels, *n* (%)						
LAD	0.821	0.474, 1.421	0.481			
RCA	0.975	0.550, 1.729	0.932			
LCX	1.401	0.690, 2.844	0.351			
LVEDD (mm)	1.090	1.039, 1.44	<0.001			
LAD (mm)	1.147	1.073, 1.226	<0.001	1.100	1.010, 1.190	0.020
LVEF (%)	0.950	0.921, 0.980	0.001			
cTnI (ng/mL)	1.001	0.993, 1.008	0.884			
CK-MB (μ/L)	1.002	1.000, 1.004	0.057			
logNT-ProBNP	2.000	1.591, 2.514	<0.001	1.520	1.130, 2.030	0.005
TG (mmol/L)	0.606	0.432, 0.851	0.004			
TC (mmol/L)	0.993	0.787, 1.254	0.955			
VLDL (mmol/L)	0.446	0.224, 0.887	0.021			
Uric acid (μmol/L)	1.003	1.000, 1.005	0.061			
Scr (μmol/L)	1.021	1.002, 1.040	0.033			
GFR (mL/min)	0.962	0.946, 0.978	<0.001			
HGB (g/L)	0.990	0.974, 1.007	0.231			
Neutrophil (10^9^/L)	1.091	1.020, 1.168	0.011			
Lymphocyte (10^9^/L)	0.771	0.514, 1.157	0.209			
Monocyte (10^9^/L)	5.692	2.976, 10.886	<0.001			
NLR	1.121	1.060, 1.195	<0.001			
PLR	1.003	1.000, 1.005	0.066			
LMR	0.352	0.219, 0.564	<0.001			
SIRI	1.249	1.159, 1.346	<0.001	1.310	1.180, 1.460	<0.001
HR (bpm)	1.019	1.003, 1.036	0.023			
SBP (mmHg)	0.992	0.978, 1.006	0.254			
DBP (mmHg)	0.976	0.955, 0.998	0.033			
Killip class ≥ II, *n* (%)	7.016	3.872, 12.747	<0.001	2.510	1.150, 5.520	0.022
Medications in-hospital, *n* (%)						
ACEI/ARB	1.033	0.593, 1.799	0.910			
Beta blocker	0.983	0.542, 1.780	0.954			
CHA_2_DS_2_-VASc score	1.378	1.186, 1.601	<0.001			

NSTEMI, non-ST segment elevation myocardial infarction; STEMI, ST segment elevation myocardial infarction; LAD, left anterior descending coronary artery; LCX, left circumflex coronary artery; RCA, right coronary artery; LVEDD, left ventricular end-diastolic diameter; LAD, left atrial diameter; LVEF, left ventricular ejection fraction; CK-MB, creatine kinase-MB; cTnI, cardiac troponin I; NT-Probnp, N-terminal pro-brain natriuretic peptide; GFR, glomerular filtration rate; HGB, hemoglobin; TC, total cholesterol; TG, triglycerides; VLDL, very low-density lipoprotein; NLR, neutrophil-tolymphocyte ratio; LMR, lymphocyte-to-monocyte ratio; PLR, platelet-to-lymphocyte ratio; SIRI, systemic inflammation response index; SBP, systolic blood pressure; DBP, diastolic blood pressure.

**Figure 2:**
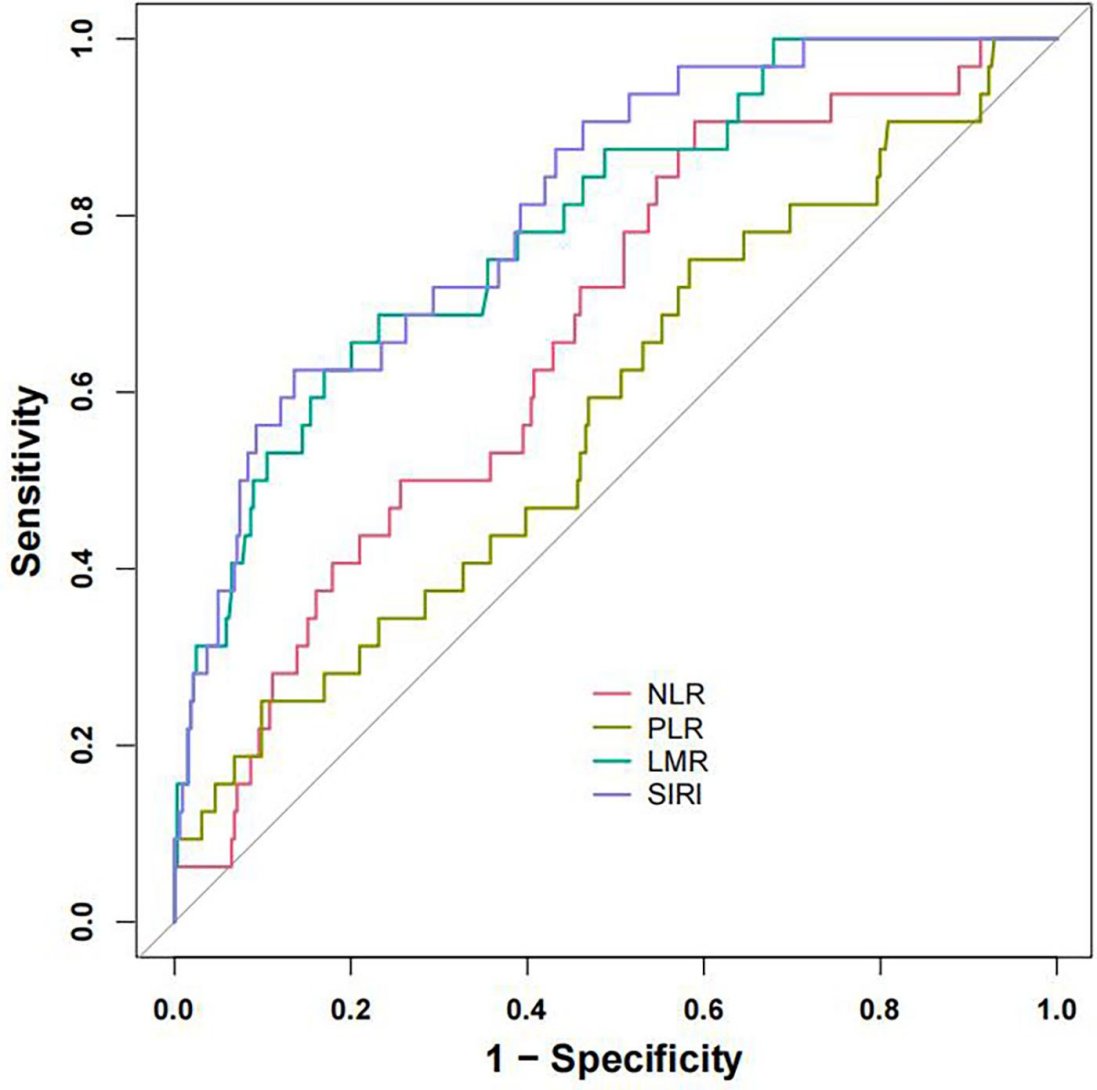
Receiver operating characteristic (ROC) curves of SIRI, LMR, NLR and PLR. ROC curves of the SIRI, LMR, NLR and PLR for predicting NOAF occurrence. ROC, receiver operating characteristic; NLR, neutrophil-tolymphocyte ratio; LMR, lymphocyte-to-monocyte ratio; PLR, platelet-to-lymphocyte ratio; SIRI, systemic inflammation response index.

ROC analyses were performed to evaluate the predictive capacity of four inflammatory markers (SIRI, LMR, NLR, and PLR) for NOAF post-PCI in AMI patients (Figure 2). Among these, SIRI and LMR demonstrated superior predictive value, with SIRI displaying the highest accuracy. The optimal cutoff value identified for SIRI in predicting NOAF occurrence after PCI in STEMI patients was 6.073, providing sensitivity and specificity of 62.5% and 86.4%, respectively (Table 3).

**Table 3:** Receiver operating characteristic (ROC) analysis results of SIRI, LMR, NLR, and PLR.

Parameter	NLR	LMR	SIRI	PLR
Cutoff value	4.667	1.641	6.073	135.443
Sensitivity	90.6%	68.8%	62.5%	75.0%
Specificity	41.0%	76.9%	86.4%	41.7%
AUC (95% CI)	0.667 (0.574–0.759)	0.792 (0.710–0.874)	0.813 (0.739–0.887)	0.580 (0.473–0.688)

AUC, area under receiver operating characteristic; NLR, neutrophil-tolymphocyte ratio; LMR, lymphocyte-to-monocyte ratio; PLR, platelet-to-lymphocyte ratio; SIRI, systemic inflammation response index; SBP, systolic blood pressure; DBP, diastolic blood pressure.

### Construction of the nomogram

**Figure 3:**
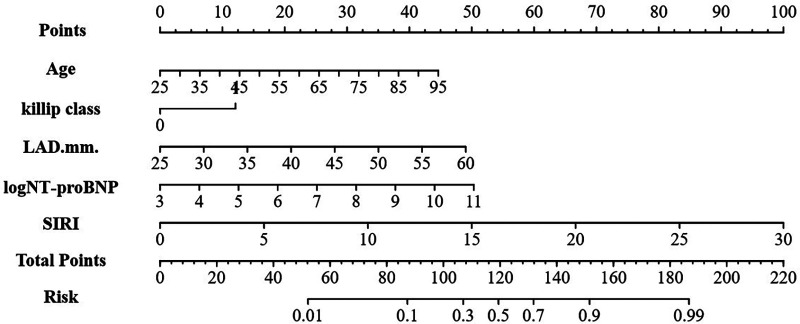
Nomogram for predicting NOAF in AMI patients after emergency PCI. LAD, left atrial diameter; SIRI, systemic inflammation response index.

A nomogram for estimating NOAF risk among AMI patients after emergency PCI was designed based on the identified independent predictors (Figure 3). Each predictor was assigned a numerical score on the horizontal axis of the nomogram, with higher total scores correlating with increased NOAF risk.

Using multivariate logistic regression findings, a practical nomogram integrating readily obtainable admission data (age, log NT-proBNP, LAD, Killip grade and SIRI) was developed for predicting the in-hospital risk of NOAF in older AMI patients (Figure 3).

### Validation of the nomogram model

**Figure 4:**
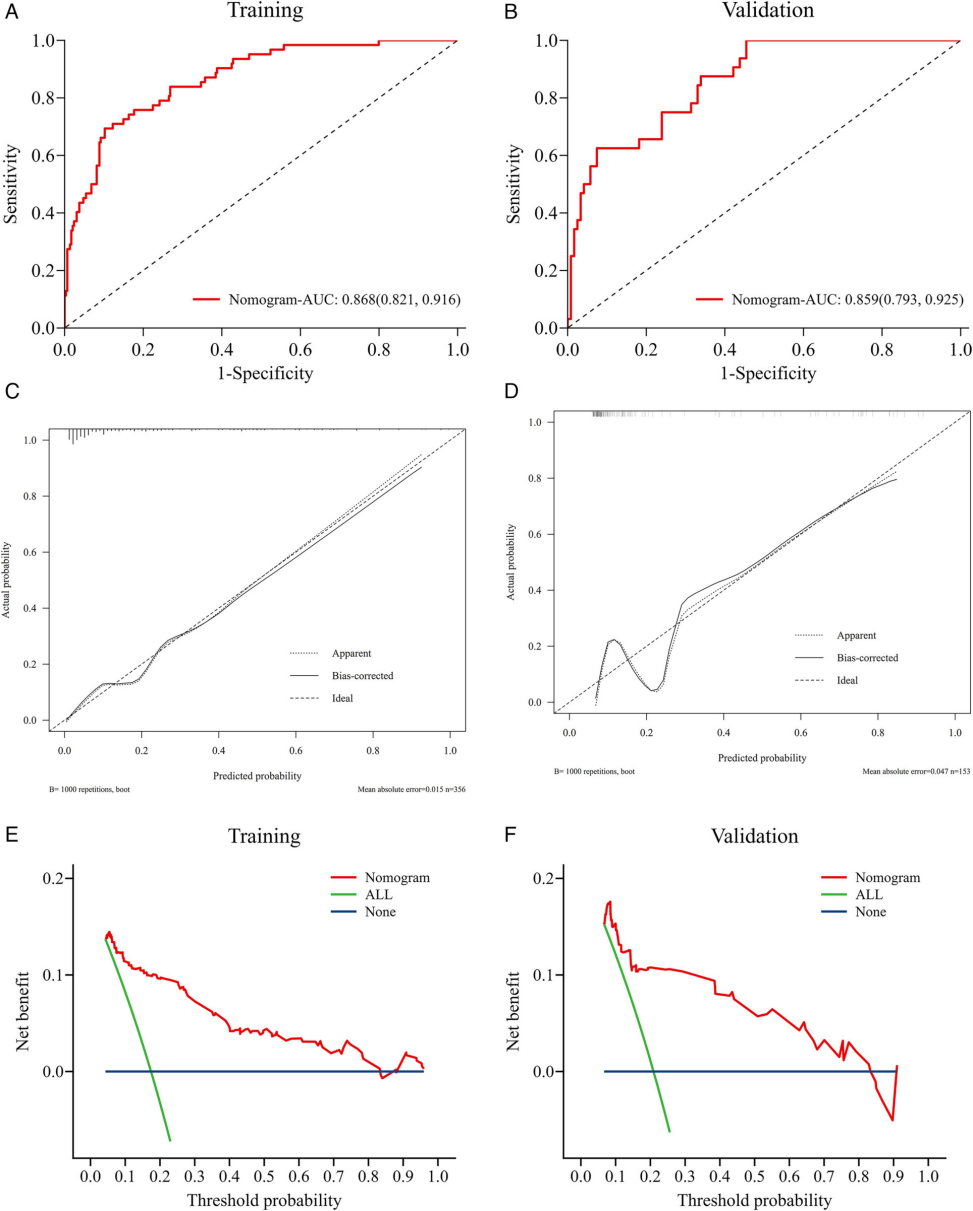
Validation of the nomogram model. Nomogram verification using training and validation cohorts [**(A,C,E)** for training cohort and **(B,D,F)** for validation cohort]. The AUCs of ROC curves are 0.868 **(A)** and 0.859 **(B)**; Both calibration plots **(C,D)** show good agreement. The DCA plots **(E,F)** suggest that the risk of in-hospital NOAF predicted by the nomogram yield net benefit in both cohorts.

**Figure 5:**
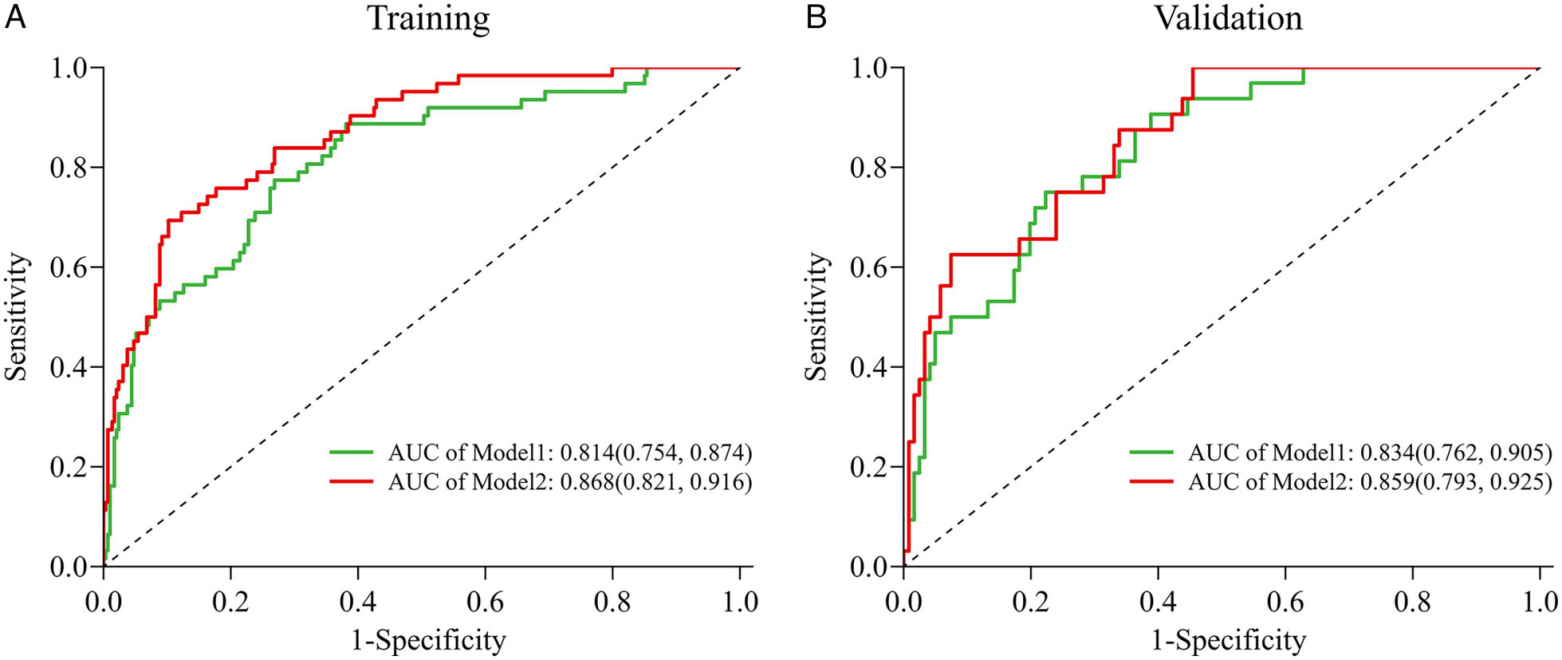
The AUC (C-index) of the nomogram with SIRI was 0.868 (95% CI: 0.821–0.916) (training cohort, **A**) and 0.859 (95% CI: 0.793–0.925) (validation cohort, **B**), demonstrating very good discrimination. The AUC (C-index) of the nomogram without Inflammatory markers was 0.814 (95% CI: 0.754–0.874) (training cohort) and 0.834 (95% CI: 0.762–0.905) (validation cohort); Model 1 = Age + Killip class + LAD + logNT-proBNP; Model2 = Age + Killip class + LAD + logNT-proBNP + SIRI. 95% CI, 95% confidence interval; AUC, areas under the curve.

We evaluated the predictive performance of the nomogram using ROC curves. Results indicated that the nomogram achieved AUCs of 0.868 (95% CI: 0.821–0.916) and 0.859 (95% CI: 0.793–0.925) in the training and validation cohorts, respectively (Figures 4A,B). The nomogram was further validated through 1,000 bootstrap resamples to generate calibration curves. These results demonstrated good agreement between the predicted probabilities and observed outcomes, with mean absolute differences of 0.015 in the training cohort and 0.045 in the validation cohort (Figures 4C,D). The Hosmer-Lemeshow (HL) tests indicated good model fit (*P* > 0.05) in both cohorts. Decision curve analysis (DCA) revealed that the nomogram provided substantial net clinical benefits within threshold probability ranges of 0.05–0.82 and 0.08–0.82 for the training and validation cohorts, respectively (Figures 4E,F). Incorporating the systemic inflammatory response index (SIRI) into the predictive model to assess NOAF risk after PCI in AMI patients may enhance clinical prognostic outcomes. Additionally, we compared the AUC values for models without SIRI in both the training and validation cohorts. Results showed that the inclusion of SIRI significantly improved the predictive ability of the nomogram, as illustrated in Figures 5A,B.

## Discussion

This retrospective study analyzed independent risk factors for postoperative NOAF among 509 AMI patients treated with PCI at Hefei First People's Hospital. Age, LAD, Killip classification, NT-proBNP, and SIRI were found to independently predict NOAF occurrence. To the authors' knowledge, this study uniquely compared the predictive effectiveness of four novel inflammatory markers (NLR, LMR, SIRI, PLR) for NOAF during hospitalization post-AMI. Results highlighted the superior predictive capability of SIRI and LMR, particularly noting the highest accuracy with SIRI (AUC: 0.813, 95% CI: 0.739–0.887). In addition, we developed a novel nomogram incorporating the systemic inflammation index. This nomogram showed strong performance (AUC: 0.868, 95% CI: 0.821–0.916) for predicting NOAF incidence after AMI. Compared to models excluding SIRI, the nomogram's predictive ability significantly improved with the inclusion of SIRI. Finally, calibration curves and DCA demonstrated excellent clinical utility of the nomogram.

Although PCI effectively restores coronary blood flow and improves patient outcomes, the risk of postoperative arrhythmias remains notable. NOAF is one of the prevalent complications following AMI, associated with extended hospital stays, higher in-hospital mortality, and worsened long-term prognosis compared to patients maintaining sinus rhythm (5, 6). The reported NOAF prevalence ranges from 6% to 21% among AMI patients (2), aligning with the observed incidence of approximately 18.5% in this investigation.

The heightened occurrence of AF in acute coronary syndrome is associated with several underlying mechanisms, such as ischemic injury, compromised or obstructed atrial circulation, elevated end-diastolic pressures within the left ventricle, and raised pressures in the left atrium (18). These pathological changes induce NOAF by initiating both structural and electrical remodeling within atrial tissues. During the acute phase of AMI, inflammatory processes at both local and systemic levels significantly contribute to cardiac repair and remodeling. Emerging evidence has highlighted the critical influence of inflammation in facilitating AF onset among AMI patients (19, 20). Key inflammatory components, including neutrophils, monocytes, platelets, and lymphocytes, directly or indirectly partake in the mechanisms leading to AF. Notably, elevated monocyte counts in AMI patients correlate with reduced left ventricular ejection fraction, increased infarction extent, and impaired microvascular perfusion. Such observations underscore the association between high pro-inflammatory monocyte counts, substantial myocardial injury, and poorer functional recovery outcomes following STEMI (21). Moreover, monocyte aggregation promotes secretion of inflammatory cytokines, which can induce atrial remodeling and subsequently AF (22). Correspondingly, elevated monocyte counts were significantly observed in NOAF patients within this study compared to those without NOAF. Neutrophils also contribute to atrial fibrosis by secreting inflammatory cytokines, proteases, and peroxidases (23). Platelets play a critical role in inflammation and thrombosis. Elevated platelet volume increases atrial and myocardial ischemia risk by altering blood viscosity and enhancing inflammation, thus increasing NOAF risk in STEMI patients (24). Conversely, lymphocytes exhibit anti-atherosclerotic properties. During MI, stress-induced lymphopenia is often associated with poorer cardiovascular outcomes (25, 26). T lymphocytes regulate innate immune responses in AF pathogenesis, while B lymphocytes may influence disease progression through autoantibody secretion. Recently, composite inflammatory indices derived from blood cell counts (NLR, LMR, PLR, SIRI) have received attention for assessing systemic inflammation and immune response in CAD, including AF. NLR and LMR have independently predicted AF (11, 12), whereas previous studies demonstrated SIRI's predictive capability for cardiovascular disease prognosis (27, 28). Additionally, a recent retrospective study involving 616 STEMI patients undergoing PCI found SIRI independently predicted NOAF and correlated positively with adverse outcomes (29). PLR, an inflammatory biomarker related to platelet aggregation, was independently associated with postoperative AF risk following CABG and AF recurrence post-radiofrequency ablation (30–32). Consistent with previous research, a recent study has found that elevated PLR independently predicted NOAF in STEMI patients post-PCI, revealing a dose-response relationship (24). Despite proposed mechanisms explaining associations between these inflammatory indices and NOAF, further research is necessary to elucidate their precise roles in AF pathogenesis. This research uniquely evaluated and compared NLR, LMR, PLR, and SIRI in predicting NOAF post-PCI in AMI patients. Findings suggested NLR, LMR, and SIRI showed robust predictive value (AUC > 0.700), with SIRI confirmed as an independent predictor through adjusted multivariate logistic regression. Specifically, the superior predictive performance of SIRI among the four indices may be attributed to its inclusion of monocytes, which represent a pivotal component in inflammatory pathways specifically linked to atrial fibrosis. Although the sensitivity of SIRI was moderate (62.5%), its high specificity (86.4%) suggests it is valuable for identifying patients truly at high risk, potentially guiding more intensive rhythm monitoring protocols. Furthermore, our findings indicated poor predictive capability of PLR for NOAF after PCI, inconsistent with previous research (24), possibly due to differences in sample size and selection criteria. Overall, NLR, LMR, and especially SIRI should be assessed upon admission in AMI patients to identify high-risk individuals requiring enhanced management and early intervention for optimal prognosis.

Inflammation is recognized as a fundamental pathophysiological driver of AF onset and progression in ACS patients. Chronic inflammatory states, particularly prevalent among elderly individuals, promote structural deterioration of atrial tissues, thereby heightening susceptibility to myocardial fibrosis and subsequent AF. The aging myocardium experiences significant anatomical and electrophysiological transformations, characterized by diminished lateral electrical connectivity among myocardial fibers and reduced conduction capabilities in cardiac structures such as the sinoatrial node, atrioventricular node, and atrial chambers (33). Earlier research has established a clear correlation between advanced age and elevated AF incidence, designating age as an essential risk determinant for AF (34). A recent retrospective cohort analysis similarly found age to be an independent predictor for NOAF among AMI patients (35). Consistently, this research observed older age among patients experiencing NOAF, further emphasizing age's role as a crucial risk factor for NOAF after PCI in AMI patients.

Killip class serves as a measure of heart failure severity following AMI, with higher classes typically associated with greater coronary artery disease burden, larger myocardial infarction areas, extensive necrosis, and subsequent fibrosis. Once established, fibrosis is difficult to reverse, impairing cardiac contractility, disrupting electrical activity, and inducing arrhythmias (36, 37). Recent studies have identified Killip class ≥III as a strong predictor of NOAF after AMI (38, 39). Our study similarly demonstrated higher Killip classes among AMI patients who developed NOAF (OR = 2.510, *P* = 0.022), consistent with previous findings.

AF and cardiac dysfunction are closely interrelated, and NT-proBNP is a traditional biomarker of clinical cardiac dysfunction. A recent meta-analysis involving 16 cohorts (8,017 AF cases) reported that including NT-proBNP in predictive models improves the early identification of AF risk (40). Our findings align with this evidence, demonstrating that elevated NT-proBNP levels increase the risk of NOAF following PCI in AMI patients.

AF results from structural and interstitial changes in myocardial cells, altering the myocardial environment. Structural remodeling, including left atrial enlargement and increased atrial fibrosis, represents a core mechanism of atrial remodeling in AF by promoting conduction disturbances (41, 42). These changes slow depolarization waves, causing electrical pulses to propagate via alternative pathways and resulting in multiple re-entry circuits (43). Recent studies consider increased LAD as an independent predictor of NOAF. Enlargement of LAD indicates progressive atrial dilation and remodeling, essential for the initiation and maintenance of AF (44). Furthermore, some scholars also reported that increased LAD, as a biomarker of atrial cardiomyopathy, is closely associated with postoperative NOAF in AMI patients (45). Consistent with their study of 4,713 AMI patients without prior AF, our results demonstrated significantly larger LAD in the NOAF group compared to the non-NOAF group.

The primary strength of this study is the construction of a visual nomogram incorporating novel systemic inflammatory markers. This nomogram demonstrated strong predictive accuracy, enabling early identification of NOAF risk in STEMI patients. It supports individualized risk assessment, superior to nomograms lacking systemic inflammatory indices. The discrimination and calibration of the nomogram were good, providing net clinical benefits in both training and validation cohorts. Compared with previous predictive models, this study offers novel insights by including systemic inflammatory indices derived from routine blood counts, which are easily obtained clinical biomarkers. Clinicians can use the systemic inflammation index upon admission to effectively identify high-risk NOAF patients, facilitating personalized treatment and improved care. Additionally, our findings support the hypothesis linking inflammation to NOAF pathogenesis, providing potential targets for NOAF prevention and treatment.

However, this study possesses several limitations. Firstly, the retrospective nature constrained patient selection to those with comprehensive medical records, potentially introducing selection biases. Secondly, the model was internally validated using a randomly split cohort from the same center, which may not fully represent external populations. Therefore, the generalizability of our nomogram requires further validation in multicenter, prospective cohorts. Thirdly, the lack of continuous ECG monitoring before hospital admission may have caused undetected pre-existing AF cases, particularly silent AF. Fourthly, not all patients received continuous ECG monitoring upon subsequent hospital admissions, possibly resulting in missed NOAF episodes. Fifthly, we did not systematically account for the potential effects of medications (e.g., statins, antibiotics, corticosteroids) on inflammatory indices, which may confound the observed associations. Lastly, the study did not include long-term patient outcomes, thereby restricting the evaluation of the identified predictors' prognostic value over extended periods. Subsequent studies should address these issues to improve understanding and reliability comprehensively.

## Conclusion

In conclusion, age, Killip class, NT-proBNP, LAD, and SIRI were identified as independent predictors of postoperative NOAF in AMI patients undergoing PCI. Among four novel inflammatory indices, SIRI and LMR demonstrated high predictive accuracy, with SIRI being the strongest predictor. The nomogram incorporating these inflammatory indices effectively predicted NOAF risk, showing good discrimination, calibration, and clinical utility. It represents an excellent tool for early NOAF risk prediction in STEMI patients post-PCI. Our findings enable clinicians to proactively assess risk and intervene before NOAF occurs, supporting the inflammatory hypothesis in NOAF pathogenesis and aiding in identifying new preventive and therapeutic targets.

## Data Availability

The datasets presented in this study can be found in online repositories. The names of the repository/repositories and accession number(s) can be found in the article/Supplementary Material.
